# Genetic and biochemical analysis of the serine/threonine protein kinases PknA, PknB, PknG and PknL of *Corynebacterium glutamicum*: evidence for non-essentiality and for phosphorylation of OdhI and FtsZ by multiple kinases

**DOI:** 10.1111/j.1365-2958.2009.06897.x

**Published:** 2009-10-13

**Authors:** Christian Schultz, Axel Niebisch, Astrid Schwaiger, Ulrike Viets, Sabine Metzger, Marc Bramkamp, Michael Bott

**Affiliations:** 1 Institut für Biotechnologie 1, Forschungszentrum JülichD-52425 Jülich, Germany; 2 Institut für Biochemie, Universität zu KölnD-50674 Köln, Germany; 3 Biologisch-Medizinisches Forschungszentrum, Heinrich-Heine-Universität DüsseldorfD-40225 Düsseldorf, Germany

## Abstract

We previously showed that the 2-oxoglutarate dehydrogenase inhibitor protein OdhI of *Corynebacterium glutamicum* is phosphorylated by PknG at Thr14, but that also additional serine/threonine protein kinases (STPKs) can phosphorylate OdhI. To identify these, a set of three single (Δ*pknA*, Δ*pknB*, Δ*pknL*), five double (Δ*pknAG*, Δ*pknAL*, Δ*pknBG*, Δ*pknBL*, Δ*pknLG*) and two triple deletion mutants (Δ*pknALG*, Δ*pknBLG*) were constructed. The existence of these mutants shows that PknA, PknB, PknG and PknL are not essential in *C. glutamicum*. Analysis of the OdhI phosphorylation status in the mutant strains revealed that all four STPKs can contribute to OdhI phosphorylation, with PknG being the most important one. Only mutants in which *pknG* was deleted showed a strong growth inhibition on agar plates containing glutamine as carbon and nitrogen source. Thr14 and Thr15 of OdhI were shown to be phosphorylated *in vivo*, either individually or simultaneously, and evidence for up to two additional phosphorylation sites was obtained. Dephosphorylation of OdhI was shown to be catalysed by the phospho-Ser/Thr protein phosphatase Ppp. Besides OdhI, the cell division protein FtsZ was identified as substrate of PknA, PknB and PknL and of the phosphatase Ppp, suggesting a role of these proteins in cell division.

## Introduction

Genome sequencing has shown that many bacterial species contain eukaryotic-like serine/threonine protein kinases (STPKs) indicating that this type of proteins plays important roles also in prokaryotic signalling and regulation (Bott, 2010). However, in the majority of cases the targets of bacterial STPKs and their specific sensory functions are still unknown. We are interested in getting a global understanding of the regulatory processes determining the physiology and metabolism of *Corynebacterium glutamicum* (Kinoshita *et al.*, 1957), a non-pathogenic Gram-positive soil bacterium that is used for large-scale industrial amino acid production (Hermann, 2003) and serves as a model organism for the suborder *Corynebacterineae* within the order *Actinomycetales*. A wealth of knowledge on this species has been summarized in two recent monographs (Eggeling and Bott, 2005; Burkovski, 2008). Whereas the majority of studies concerning regulation in *C. glutamicum* cope with transcriptional regulation (for an overview see Brinkrolf *et al.*, 2007), only few are dealing with regulation at the post-transcriptional level such as conditional proteolysis (Engels *et al.*, 2004; 2005; Strösser *et al.*, 2004) or regulation by STPKs (Niebisch *et al.*, 2006; Schultz *et al.*, 2007; Fiuza *et al.*, 2008).

The *C. glutamicum* genome sequence (Ikeda and Nakagawa, 2003; Kalinowski *et al.*, 2003) has unravelled the presence of four genes encoding eukaryotic-like STPKs, designated PknA (cg0059), PknB (cg0057), PknG (cg3046) and PknL (cg2388). In addition, a single gene (*ppp*, cg0062) coding for a phospho-serine/threonine protein phosphatase was annotated. In a recent study we analysed the function of PknG (Niebisch *et al.*, 2006). A *pknG* deletion mutant had a strong defect in utilizing l-glutamine as carbon, energy and nitrogen source. A proteome comparison of wild type and Δ*pknG* mutant followed by *in vitro* phosphorylation studies led to the identification of the 15 kDa OdhI (2-oxoglutarate dehydrogenase inhibitor) protein as a target of PknG. OdhI (143 amino acid residues) is composed of an N-terminal domain of 42 amino acid residues which is followed by an FHA domain (residues 43–143) (Barthe *et al.*, 2009). Forkhead-associated (FHA) domains are known to bind to phosphothreonine epitopes of proteins (Pallen *et al.*, 2002; Liang and Van Doren, 2008). PknG phosphorylates OdhI at threonine residue 14 (Niebisch *et al.*, 2006). When native OdhI was replaced by an OdhI-T14A mutein, the corresponding strain showed a similar phenotype as the Δ*pknG* mutant, indicating that phosphorylation of OdhI at T14 is required for glutamine utilization and that unphosphorylated OdhI is inhibitory for glutamine utilization. In line with this interpretation, deletion of the *odhI* gene in the Δ*pknG* mutant suppressed the growth defect on glutamine.

In a search for protein interaction partners, unphosphorylated OdhI was found to bind specifically to the E1 subunit OdhA of the 2-oxoglutarate dehydrogenase complex (ODHc). Enzyme assays revealed that unphosphorylated OdhI inhibits the activity of ODHc and phosphorylation by PknG relieves this inhibition. As ODHc is required for glutamine utilization, the growth defects of the Δ*pknG* mutant and the strain carrying OdhI-T14A are probably due to the inhibition of ODHc by OdhI (Niebisch *et al.*, 2006). The presence of OdhI is of key importance for the production of l-glutamate, the major amino acid (1.5 million tons per year) produced with *C. glutamicum* (Schultz *et al.*, 2007).

Interestingly, two-dimensional (2D) gel electrophoresis of protein extracts from wild-type cells revealed the presence of three OdhI protein spots of similar molecular mass but different isoelectric point (pI) (Niebisch *et al.*, 2006). This indicated that OdhI can exist not only in a monophosphorylated state, but also in a diphosphorylated state. Support for this assumption was obtained from the fact that in the Δ*pknG* mutant the diphosphorylated form was absent, but a small fraction of OdhI still migrated as monophosphorylated form. Further evidence for the assumption that one or several other STPKs besides PknG can phosphorylate OdhI *in vivo* was obtained by Western blot analysis with polyclonal OdhI antibodies (Schultz *et al.*, 2007). Recently, the purified kinase domains of PknA and PknB were shown to phosphorylate OdhI *in vitro* (Fiuza *et al.*, 2008) and T15 was identified as phosphorylation site of these two kinases (Barthe *et al.*, 2009). Replacement of the native OdhI protein by an OdhI-T15A mutein did not lead to a strong growth defect on glutamine plates, in contrast to the OdhI-T14A mutein (Niebisch *et al.*, 2006). The basis for this difference is not yet clear.

In *Mycobacterium tuberculosis*, the GarA protein, which shares 69% sequence identity to OdhI, was found to be the best detectable *in vitro* substrate of the PknB kinase domain, which phosphorylates GarA at T22, which corresponds to T15 in OdhI (Villarino *et al.*, 2005). PknG of *M. tuberculosis* was shown to phosphorylate GarA at T21, which corresponds to T14 in OdhI (O'Hare *et al.*, 2008). Similar to the situation in *C. glutamicum*, unphosphorylated GarA was found to inhibit the 2-oxoglutarate decarboxylase activity of the OdhA homologue SucA (O'Hare *et al.*, 2008). In *M. tuberculosis*, no ODHc activity could be detected despite the presence of SucA, SucB and Lpd and an alternative pathway was proposed in which SucA decarboxylates 2-oxoglutarate to succinate semialdehyde which then is converted by succinate semialdehyde dehydrogenase to succinate (Tian *et al.*, 2005a,b;). Besides SucA, unphosphorylated GarA also inhibited a second enzyme, an NAD^+^-dependent glutamate dehydrogenase (O'Hare *et al.*, 2008), which is absent in *C. glutamicum*. Recently, biochemical studies led to the proposal that GarA functions as a molecular switch. Evidence was provided indicating that the phosphorylated N-terminal domain is bound by the C-terminal FHA domain of GarA, leading to a blockage of this domain and an altered conformation of the protein (England *et al.*, 2009). Such an altered conformation is in line with the observation that unphosphorylated and phosphorylated OdhI show a distinct migration behaviour even in denaturing SDS-polyacrylamide gel electrophoresis (Niebisch *et al.*, 2006; Schultz *et al.*, 2007). Barthe *et al.* (2009) recently determined by NMR the solution structures of unphosphorylated OdhI and OdhI phosphorylated by PknB on T15. These structures confirmed the molecular switch model first proposed by England *et al.* (2009): upon phosphorylation, a large conformational change takes place and the N-terminal part of the protein is bound by its own FHA domain via the phosphorylated threonine residue, thereby blocking the binding of the FHA domain to other interaction partners.

In the present study, we analysed the role of PknA, PknB and PknL in the phosphorylation of OdhI, both *in vivo* and *in vitro*. For this purpose, a series of single, double and triple STPK deletion mutants were constructed and analysed for growth and the phosphorylation status of OdhI. Additionally, the kinase domains of all four STPKs and the cytoplasmic domain of Ppp were purified and tested for OdhI phosphorylation and OdhI dephosphorylation *in vitro* respectively. Finally, an important cell division protein, FtsZ, was identified as a novel substrate of STPKs in *C. glutamicum*.

## Results

### Genomic organization of *pknA*, *pknB*, *pknG* and *pknL* in *C. glutamicum* and domain structure of the corresponding proteins

**Fig. 1:**
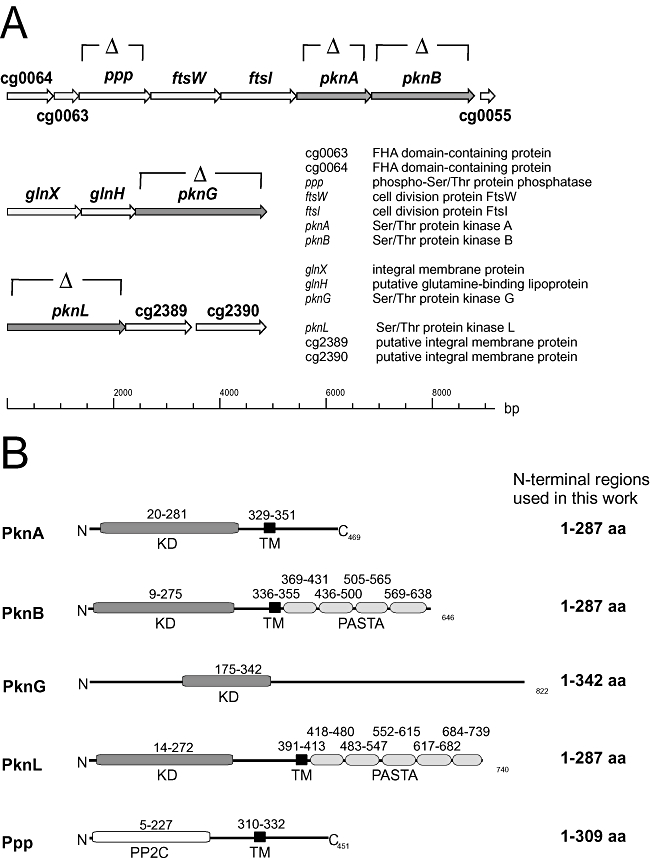
Genomic organization of *pknA*, *pknB*, *pknG*, *pknL* and *ppp* in *C. glutamicum* (A) and domain architecture of the corresponding proteins as predicted by PFAM, TMHMM or TMPred (B). KD, kinase domain; TM, transmembrane helix; PP2C, phosphatase domain; PASTA, penicillin-binding protein and serine/threonine kinase-associated domain.

In the genome of *C. glutamicum* four STPKs and only a single phospho-serine/threonine protein phosphatase have been annotated (Ikeda and Nakagawa, 2003; Kalinowski *et al.*, 2003). The genomic organization is shown in Fig. 1A. PknA (469 aa), PknB (646 aa) and Ppp (451 aa) are encoded in a putative operon together with five other genes, encoding two FHA domain-containing proteins (cg0063, cg0064), the cell division proteins FtsW (cg0061) and FtsI (cg0060), and a hypothetical membrane protein (cg0055). The gene encoding PknG (822 aa) is clustered in a putative operon with *glnX* and *glnH* encoding a membrane protein and a putative glutamine-binding lipoprotein respectively (Niebisch *et al.*, 2006). The gene encoding PknL (740 aa) is located upstream of cg2389 and cg2390, both encoding hypothetical membrane proteins.

In Fig. 1B, the domain architecture of the four STPKs and Ppp is shown as predicted by analysis with Pfam (http://pfam.janelia.org) and TMHMM (http://www.cbs.dtu.dk/services/TMHMM) or TMPred (http://www.ch.embnet.org/software/TMPRED_form.html). Whereas PknG is a soluble protein, PknA, PknB and PknL each contain a single transmembrane helix extending approximately from amino acid residues 329 to 351 (PknA), 336 to 355 (PknB) and 391 to 413 (PknL). Hence, PknA, PknB and PknL are probably membrane-integral proteins. The kinase domains of PknA, PknB and PknL are located in the N-terminal, cytoplasmically located portions of the proteins. In the C-terminal, extracytoplasmic parts of PknB and PknL, four and five so-called PASTA domains were identified which are involved in binding of peptidoglycan components (Yeats *et al.*, 2002; Shah *et al.*, 2008). The phosphatase Ppp also contains a single transmembrane helix (residues 310–332) and the phosphatase domain of the PP2C family is located in the N-terminal cytoplasmic portion of the protein.

### Construction of in-frame deletion mutants provides evidence for the non-essentiality of all STPKs in *C. glutamicum*

To analyse *in vivo* the influence of the different STPKs on the phosphorylation status of OdhI and other potential target genes, a series of three single (Δ*pknA*, Δ*pknB*, Δ*pknL*), five double (Δ*pknAG*, Δ*pknAL*, Δ*pknBG*, Δ*pknBL*, Δ*pknLG*) and two triple deletion mutants (Δ*pknALG* and Δ*pknBLG*) were constructed as described in *Experimental procedures*. In Fig. S1A, the verification by polymerase chain reactions (PCR) of the chromosomal deletions in the different mutants is shown. Strains Δ*pknA* and Δ*pknB* were additionally analysed by Southern blot analysis, which again confirmed the deletion of the corresponding genes (Fig. S1B). The fact that we were able to obtain all of these mutants clearly demonstrates that none of the four STPK genes of *C. glutamicum* is essential under the conditions tested. The only STPK double deletion mutant which we could not obtain was the one lacking *pknA* and *pknB*, suggesting a case of conditional lethality.

### Growth properties and cell morphology of the deletion mutants

**Fig. 2:**
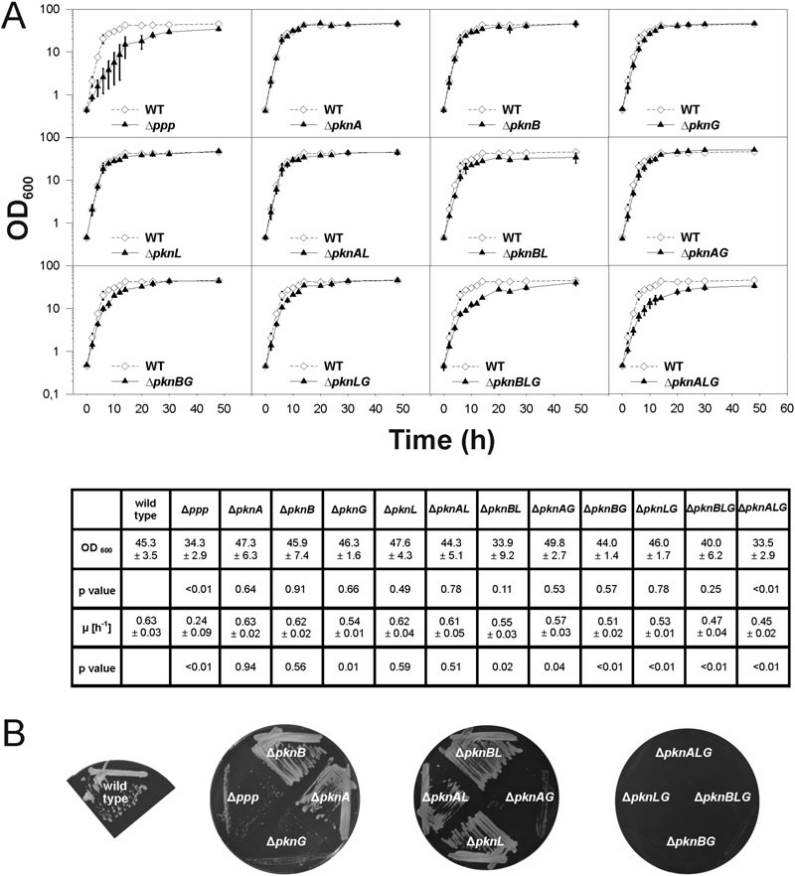
Growth of STPK and Ppp mutants of *C. glutamicum*. A. Growth of different *C. glutamicum* mutants (black symbols) in comparison with the wild type (white symbols) in BHI medium containing 4% (w/v) glucose. Mean values of triplicate experiments are shown. The final optical densities at 600 nm (OD_600_) with standard deviations and the maximal growth rates are listed below as well as the *P*-values from a *t*-test for pairwise comparisons of the mutants with the wild type. B. Growth on CGXII agar plates containing 100 mM l-glutamine as sole carbon and nitrogen source.

In Fig. 2A, growth curves of the different STPK deletion mutants and the Δ*ppp* mutant in BHI medium with 4% (w/v) glucose in comparison with the wild type are shown as well as final optical densities at 600 nm (OD_600_) and growth rates that were calculated from three independent experiments. The strongest growth defect was observed for the Δ*ppp* mutant and the two triple deletion mutants Δ*pknALG* and Δ*pknBLG*. Slightly reduced growth rates were observed for the double deletion mutants except for strain Δ*pknAL* and a significantly reduced final OD_600_ was measured for the Δ*pknBL* mutant. In the case of the single deletion mutants, only the Δ*pknG* mutant showed a slightly reduced growth rate. These results confirm the non-essentiality of all STPKs in *C. glutamicum*, but also show that the simultaneous deletion of two or three of these kinases has negative effects on growth even in a rich complex medium.

In our previous study we showed that the Δ*pknG* mutant is strongly impaired in its ability to grow on minimal medium agar plates containing l-glutamine as sole carbon and nitrogen source (Niebisch *et al.*, 2006). We therefore tested growth of the other mutants on this medium. As shown in Fig. 2B, only the mutants lacking *pknG* were strongly impaired in glutamine utilization, whereas the single and double mutants with an intact *pknG* gene were able to grow, i.e. strains Δ*pknA*, Δ*pknB*, Δ*pknL*, Δ*pknAL* and Δ*pknBL*.

**Fig. 3:**
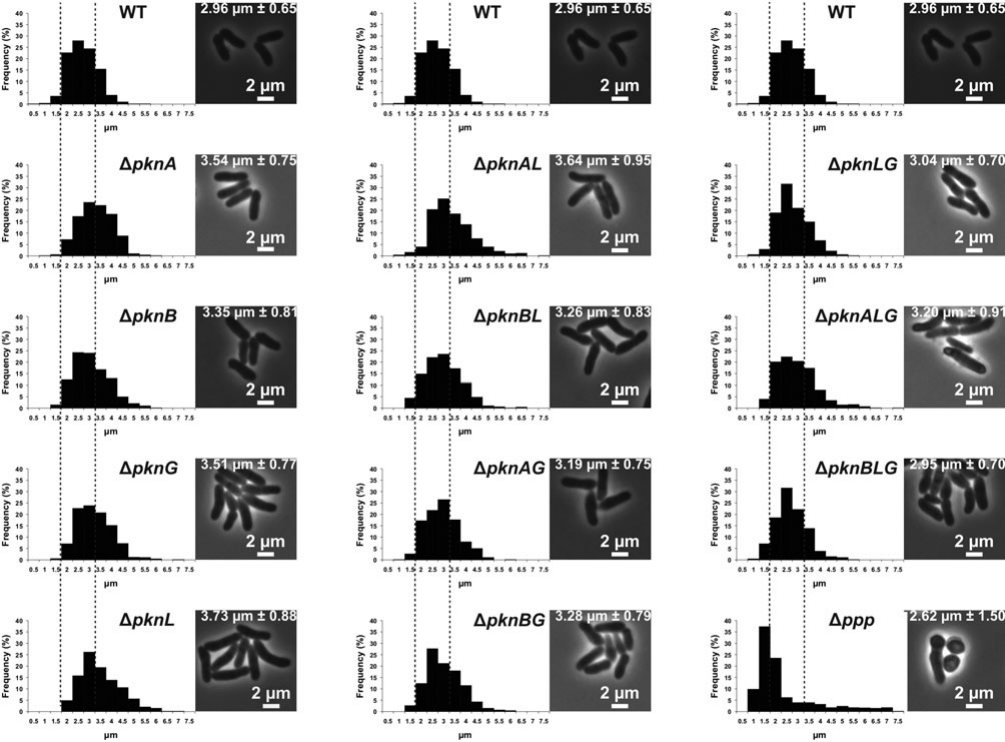
Cell morphology of STPK and Ppp mutants of *C. glutamicum* growing in BHI medium with 4% (w/v) glucose at 30°C (early exponential phase) was observed. Phase-contrast microscopy pictures are shown and 500 cells of each strain were counted to determine the frequency of cell length. The dotted lines represent the length range from 1.5 to 3 μm which covers 75% of the wild-type cells.

To further characterize the different mutant strains, cell morphology was analysed by phase-contrast microscopy after growth in BHI medium containing 4% (w/v) glucose. As shown in Fig. 3, all STPK mutant cells were elongated in comparison with the wild type except for the triple deletion strain Δ*pknBLG*. These results suggest that the STPKs of *C. glutamicum* are involved in cell morphogenesis and possibly cell division. In contrast, the *ppp* deletion strain showed a highly abnormal and pleomorphic cell morphology. The majority of cells were shortened and often had bulged cell poles, whereas a minority was strongly elongated.

### *In vivo* analysis of the OdhI phosphorylation status

**Fig. 4:**
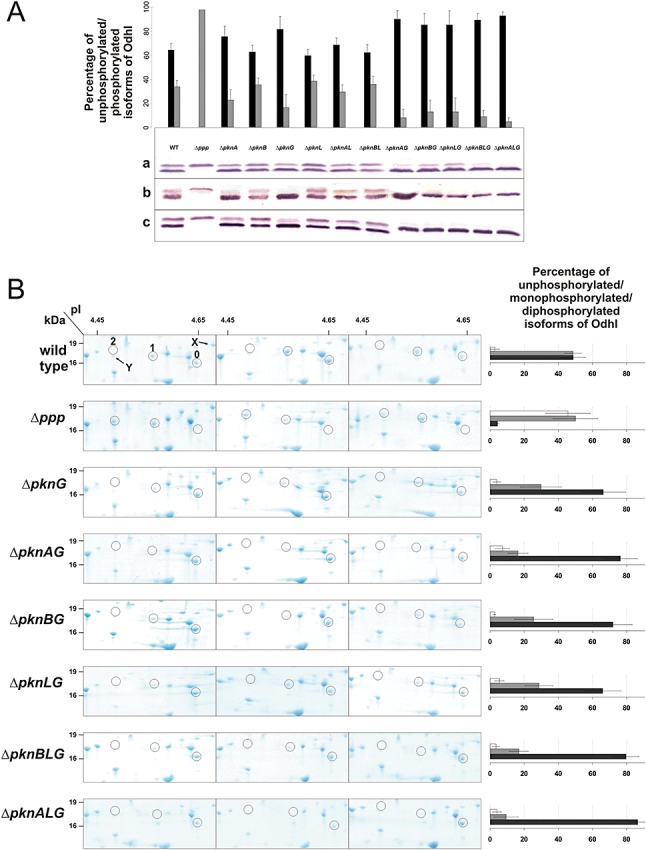
Analysis of the *in vivo* OdhI phosphorylation status in cells grown for 24 h in BHI medium with 4% (w/v) glucose. A. Western blot analysis of cell-free protein extracts (20 μg each) of the indicated *C. glutamicum* strains with OdhI antibodies. The experiment was performed in triplicate (a, b, c). The upper band in the Western blot represents singly or doubly phosphorylated OdhI, the lower band unphosphorylated OdhI (Schultz *et al.*, 2007). The percentages (mean values with standard deviation) of unphosphorylated OdhI (black bars) to phosphorylated OdhI (grey bars) were calculated by densitometry. B. Two-dimensional polyacrylamide gel electrophoresis of cell-free protein extracts (300 μg each) of the indicated *C. glutamicum* strains. In the left part of the figure, sections of three 2D gels prepared with extracts of three independent cultures of the respective strain are shown. The three spots representing unphosphorylated, monophosphorylated and presumably diphosphorylated OdhI are circled and labelled with 0, 1 and 2 respectively. The relative amounts of the three OdhI forms were calculated by densitometric analysis of the OdhI spots. Normalization was performed using the spot labelled X that represents adenylate kinase (cg0648), a protein whose intracellular level is apparently not influenced by the deletion of STPKs or Ppp. The percentage of unphosphorylated OdhI is shown with black bars, that of monophosphorylated OdhI with grey bars, and that of presumably diphosphorylated OdhI by white bars. The spot labelled Y represents MenG (*S*-adenosylmethionine:2-demethylmenaquinone methyltransferase, cg1055) and is shown because of the small distance to the OdhI spot labelled 2.

We recently showed that OdhI is phosphorylated *in vivo* by PknG, but also obtained evidence for OdhI phosphorylation by one or several of the other STPKs (Niebisch *et al.*, 2006; Schultz *et al.*, 2007). With the mutants described above we tested which of the STPKs PknA, PknB and PknL is responsible for PknG-independent phosphorylation *in vivo*. In a first approach, we used a Western blot assay with OdhI antibodies to visualize the OdhI phosphorylation status in the different mutant strains after growth in BHI medium with 4% (w/v) glucose (Fig. 4A). This assay, which was performed in triplicate, can distinguish between unphosphorylated and phosphorylated OdhI, but not between mono- and diphosphorylated OdhI (Schultz *et al.*, 2007). In a second approach, the OdhI phosphorylation status of selected mutants was analysed by 2D gel electrophoresis (Fig. 4B). This method allows to distinguish between unphosphorylated, monophosphorylated and presumably diphosphorylated OdhI (Niebisch *et al.*, 2006). The wild type and mutants Δ*ppp*, Δ*pknG*, Δ*pknAG*, Δ*pknBG*, Δ*pknLG*, Δ*pknALG* and Δ*pknBLG* were tested in triplicate using independent cultures grown in BHI medium with 4% (w/v) glucose. The spots representing OdhI were confirmed by peptide mass fingerprinting. The Western blots and the 2D gels were quantitatively evaluated by densitometry and the results are given as percentage of unphosphorylated and phosphorylated OdhI in Fig. 4and Table S1. Both methods yielded comparable results. In the wild type, about 35–50% of OdhI was present in the phosphorylated state and in the Δ*ppp* mutant almost 100%. PknG was identified as the kinase primarily responsible for OdhI phosphorylation, as all mutants lacking the *pknG* gene showed a clearly diminished OdhI phosphorylation level. Besides PknG, PknA was most important for OdhI phosphorylation and PknB and PknL played only minor roles under the chosen conditions. However, caution has to be taken in the interpretation of the effects of the kinase deletions as the kinases may potentially phosphorylate each other and thus deletion of one kinase could affect the activity of another.

### Mass spectrometric analysis of the *in vivo* OdhI phosphorylation sites: evidence for the simultaneous phosphorylation of T14 and T15 and additional phosphorylation sites

**Fig. 5:**
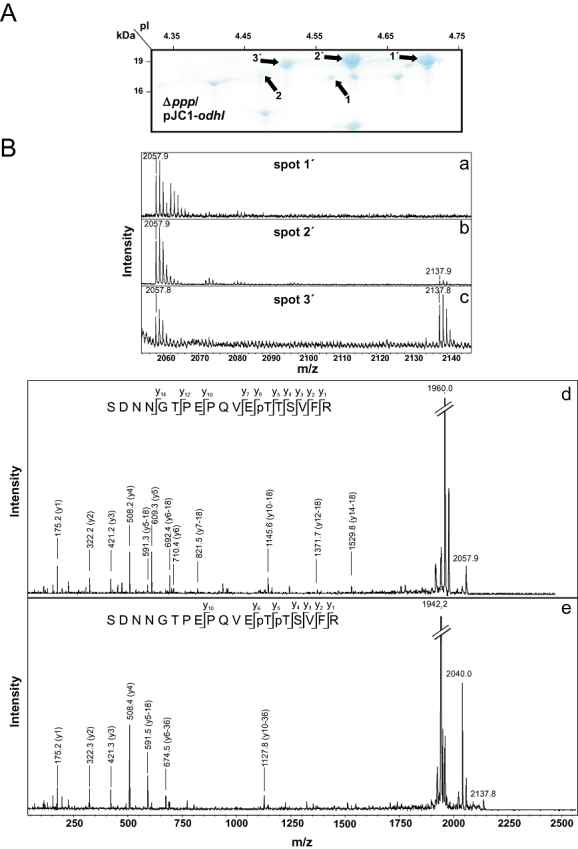
Identification of *in vivo* phosphorylation sites of OdhI. A. Analysis of the *in vivo* OdhI phosphorylation status by 2D gel electrophoresis of cell-free protein extract of *C. glutamicum*Δ*ppp*/pJC1-*odhI*. The strain was cultivated for 24 h in BHI medium with 4% (w/v) glucose and 300 μg of protein was used for separation by 2D-PAGE. The two spots representing native monophosphorylated and diphosphorylated OdhI are labelled as 1 and 2, respectively, and *Strep*-tagged monophosphorylated, diphosphorylated and presumably triphosphorylated OdhI are labelled with 1′, 2′ and 3′ respectively. B. MALDI-TOF-MS and MS/MS analysis of tryptically digested OdhI spots 1′ (panel a), 2′ (panel b) and 3′ (panel c) of strain *C. glutamicum*Δ*ppp*/pJC1-*odhI*. Peaks are labelled with their monoisotopic masses. The only phosphopeptide detected was the N-terminal one composed of amino acids 2–19. In the unphoshorylated state, the predicted mass (in the H^+^ form) is 1977.9 Da, in the monophosphorylated state 2057.9 Da, in the diphosphorylated state 2137.9 Da. In panels d and e, MALDI-TOF tandem MS of the 2057.9 Da peptide derived from spot 2′ and of the 2137.8 Da peptide derived from spot 3′ are shown respectively. β-Elimination of phosphoric acid (mass shifts of −18 Da or −36 Da compared to the unphosphorylated fragment after a single or a double β-elimination respectively) is indicated.

In this and our previous study (Niebisch *et al.*, 2006), three OdhI spots were detectable by 2D gel analysis in the *C. glutamicum* wild type and proposed to represent the non-phosphorylated, singly phosphorylated and doubly phosphorylated forms of OdhI. To support this proposal, we analysed the spots by MALDI-TOF/TOF-MS and could detect a peptide with a mass of 2057.9 Da in the presumed monophosphorylated OdhI spot of wild-type extracts (data not shown). This mass corresponds to the phosphorylated form of the N-terminal tryptic peptide of OdhI covering amino acids 2–19 (S_2_DNNGT_7_PEPQVET_14_T_15_S_16_VFR), which has a predicted mass of 1977.9 Da (note that the N-terminal methionine is absent in the native protein, nevertheless we stick to our previous numbering which includes this residue). In the case of the presumed doubly phosphorylated OdhI spot we were not successful in identifying phosphorylated peptides, presumably due to insufficient material. Therefore, we transferred plasmid pJC1-*odhI* (Niebisch *et al.*, 2006) into the *C. glutamicum*Δ*ppp* mutant and prepared 2D gels of this strain after growth for 24 h in BHI medium with 4% (w/v) glucose. The OdhI protein encoded by pJC1-*odhI* contains a C-terminal *Strep*Tag-II (WSHPQFEK) which increases the mass of the protein by about 1 kDa and the theoretical pI is shifted by 0.1–0.2 pH units towards the alkaline range. As shown in Fig. 5A, three new spots, labelled 1′, 2′ and 3′, were detectable in strain Δ*ppp*/pJC1-*odhI*, all of which were identified as OdhI by peptide mass fingerprinting. In spot 1′, a peptide with a mass of 2057.9 Da was detected, representing the singly phosphorylated N-terminal peptide (amino acids 2–19) (Fig. 5B, panel a). In spot 2′, a peptide with the same mass was present, too, but in addition a peptide of 2137.8 Da, representing a doubly phosphorylated form of the N-terminal peptide (Fig. 5B, panel b). In spot 3′, both the mono- and the diphosphorylated peptide were detected with comparable signal intensities (Fig. 5B, panel c). MS/MS analysis of the 2057.9 Da peptide (Fig. 5B, panel d) indicated that predominantly T14 is phosphorylated, but to some extent also T15 [estimated from the ratio of the signal intensities of the phosphorylated y_6_ fragment after β-elimination of phosphoric acid (692.4 Da) to the unphosphorylated y_6_ fragment (710.4 Da) and from the ratio of the phosphorylated y_5_ fragment after β-elimination of phosphoric acid (591.3 Da) to the unphosphorylated y_6_ fragment (609.3 Da)]. MS/MS of the 2137.8 Da peptide (Fig. 5B, panel e) provided evidence for the simultaneous phosphorylation of T14 and T15. In this spectrum a y_5_ fragment of m/z 591.5 was detected and a y_6_ fragment of m/z 674.5. The latter fragment is expected after two β-elimination reactions from simultaneously phosphorylated T14 and T15, which causes a reduction of the y_6_ mass by 36 Da. In addition, the existence of the doubly phosphorylated peptide is confirmed by a fragment of 1942.2 Da, which corresponds to the full-length peptide (2137.8 Da) after two β-elimination steps (mass shift of −196 Da).

The results described above confirm the existence of doubly phosphorylated OdhI and indicate that besides T14 and T15 up to two additional phosphorylation sites exist in OdhI. According to the MS data and based on the fact that unphosphorylated OdhI is absent in the Δ*ppp* mutant, the three OdhI spots observed in strain Δ*ppp*/pJC1-*odhI* represent singly, doubly and triply phosphorylated OdhI. The presence of the singly phosphorylated N-terminal peptide in spot 2′ and 3′ argues in favour of the existence of one and two additional phosphorylation sites in the residual portion of OdhI respectively. As the MALDI-TOF-MS data did not uncover these predicted additional phosphorylation sites, alternative MS methods will be applied for their identification.

### *In vitro* phosphorylation studies

**Fig. 6:**
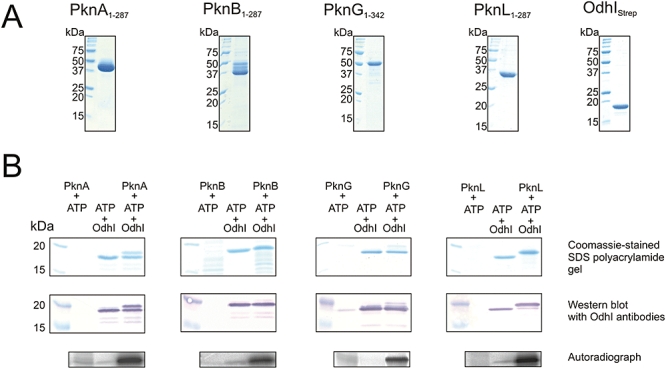
*In vitro* phosphorylation of OdhI by different STPKs. A. Kinase domains purified for *in vitro* analysis. PknA_1−287_ (34.0 kDa), PknB_1−287_ (33.4 kDa), PknG_1−342_ (40.1 kDa) and PknL_1−287_ (33.6 kDa), all containing an N-terminal decahistidine tag, were overproduced in *E. coli* and purified by Ni^2+^-chelate affinity chromatography. OdhI_Strep_ (16.6 kDa) containing a C-terminal *Strep*Tag-II was overproduced in *E. coli* and purified by *Strep*Tactin affinity chromatography. Purified proteins were subjected to SDS-PAGE and stained with Coomassie brilliant blue. B. *In vitro* phosphorylation of OdhI by PknA_1−287_, PknB_1−287_, PknG_1−342_ and PknL_1−287_. The *in vitro* phosphorylation assays were performed as described in *Experimental procedures* either with non-radioactive ATP or with [γ-^33^P]-ATP. In the former case, the OdhI phosphorylation status was followed by Coomassie-stained SDS gels and by Western blot analysis with OdhI antibodies. In the latter case, the samples were subjected to autoradiography. Only the OdhI section is shown.

In order to complement the *in vivo* studies, we also analysed phosphorylation of OdhI *in vitro*. For this purpose, OdhI was overproduced in *Escherichia coli* and purified by means of a C-terminal *Strep*Tag-II (Fig. 6A). The N-terminal portions of PknA, PknB, PknG and PknL encompassing the catalytic kinase domain were overproduced in *E. coli* and purified by means of an N-terminal His-tag (Fig. 6A). All kinase proteins including PknG_1−342_ showed autophosphorylation activity (data not shown). The ability of PknA_1−287_, PknB_1−287_, PknG_1−342_ and PknL_1−287_ to phosphorylate OdhI was analysed by Coomassie-stained SDS gels, by Western blot analysis with OdhI antibodies, and by autoradiography of assays including [γ-^33^P]-ATP. As shown in Fig. 6B, all tested kinase domains were able to phosphorylate OdhI. Barthe *et al.* (2009) reported that the kinase domains of PknA and PknB phosphorylate T15 of OdhI *in vitro*. Our MALDI-TOF-MS analysis of OdhI phosphorylated by PknL_1−287_ showed that again the N-terminal tryptic peptide covering amino acids 2–19 is phosphorylated (peptide mass 2057.9 Da), but did not allow a conclusive identification of the phosphorylation site.

In a recent study it was proposed that PknG is incapable of autophosphorylation and requires PknA for phosphorylation (Fiuza *et al.*, 2008). The fact that we were able to show autophosphorylation of full-length PknG in our recent study (Niebisch *et al.*, 2006) was explained by the assumption that PknG was purified from *C. glutamicum* wild type and presumably already phosphorylated by PknA. In order to test this assumption, we purified full-length PknG_Strep_ from the Δ*pknA* mutant. As shown in Fig. S2, the purified PknG_Strep_ showed autophosphorylation activity and was capable of phosphorylating OdhI.

### *In vitro* dephosphorylation of OdhI by the phosphatase Ppp

**Fig. 7:**
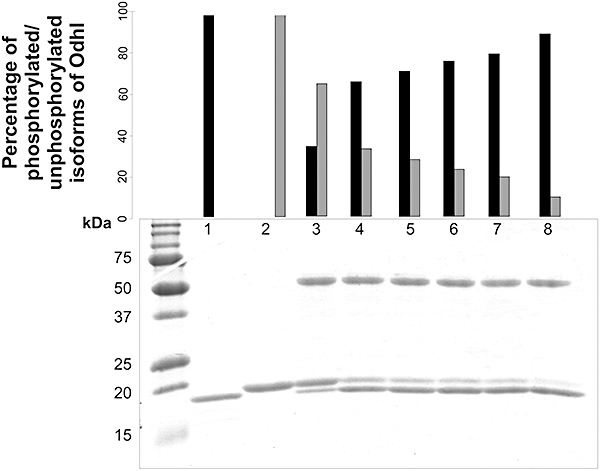
*In vitro* dephosphorylation of phosphorylated OdhI by Ppp. Ppp_1−309_ (34.7 kDa) containing an N-terminal decahistidine tag was overproduced in *E. coli* and purified by Ni^2+^-chelate affinity chromatography. The *in vitro* dephosphorylation assay was performed as described in *Experimental procedures*. Lane 1, unphosphorylated OdhI_Strep_ (purified from *E. coli* BB1553/pAN3K-odhI); lane 2, phosphorylated OdhI_Strep_ (purified from *C. glutamicum*Δ*ppp*/pJC1-*odhI*); lane 3–8, phosphorylated OdhI_Strep_ 1, 5, 10, 15, 30 and 120 min after addition of the phosphatase Ppp_1−309_. The samples were subjected to SDS-PAGE and stained with Coomassie brilliant blue. The upper band represents singly or doubly phosphorylated OdhI, the lower band unphosphorylated OdhI (Schultz *et al.*, 2007). The percentage of unphosphorylated (black bars) and phosphorylated OdhI (grey bars) was calculated by densitometric analysis.

Our previous results (Schultz *et al.*, 2007) and those reported above show that in the Δ*ppp* mutant OdhI is only present in the phosphorylated state. We interpreted this result by the assumption that the Ppp protein is responsible for the dephosphorylation of phosphorylated OdhI. To confirm this assumption, we overproduced the cytoplasmic phosphatase domain (amino acids 1–309) of Ppp in *E. coli* and purified the protein by means of an N-terminal histidine tag. Phosphorylated OdhI containing a C-terminal *Strep*Tag-II was purified by *Strep*Tactin affinity chromatography from *C. glutamicum* strain Δ*ppp* carrying plasmid pJC1-*odhI*. As shown in Fig. 7, the phosphatase domain of Ppp catalysed the dephosphorylation of OdhI, confirming that OdhI is a substrate of Ppp.

### Identification of FtsZ as a substrate of PknA, PknB and PknL in *C. glutamicum*

**Fig. 8:**
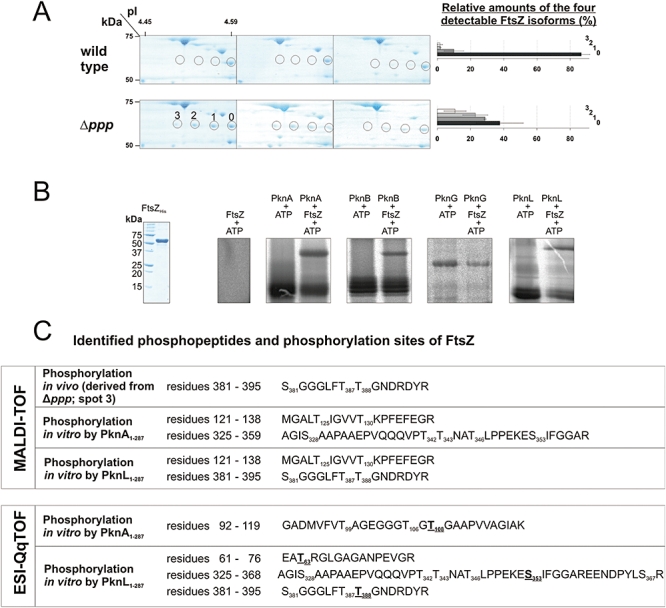
Identification of FtsZ as an *in vivo* substrate of Ppp and as an *in vitro* substrate for PknA_1−287_, PknB_1−287_ and PknL_1−287_. A. Analysis of the FtsZ phosphorylation status *in vivo* by 2D gel electrophoresis of cell-free extracts of *C. glutamicum* wild type and *C. glutamicum*Δ*ppp*. Both strains were cultivated for 24 h in BHI medium with 4% (w/v) glucose and 300 μg of protein of cell-free extracts was used for separation by 2D-PAGE. For isoelectric focusing, IPG strips (GE Healthcare) with a pH gradient from 4.0 to 5.0 were used, for subsequent SDS-PAGE Excel SDS gradient gels 12–14% (GE Healthcare). In the left part of the figure, sections of three 2D gels prepared from cell extracts of three distinct cultures of the respective strain are shown. The four spots representing presumably unphosphorylated, monophosphorylated, diphosphorylated and triphosphorylated FtsZ are circled and labelled with 0, 1, 2 and 3 respectively. The relative amounts of the four detectable FtsZ isoforms were calculated by densitometric analysis of the FtsZ spots (spot 0, black bars; spot 1, dark grey bars; spot 2, light grey bars; spot 3, white bars). B. Full-length FtsZ (amino acid residues 1–442; 49.7 kDa) containing an N-terminal His tag was overproduced in *E. coli* and purified by Ni^2+^-chelate affinity chromatography. A sample was subjected to SDS-PAGE and stained with Coomassie brilliant blue. Purified FtsZ was incubated with PknA_1−287_, PknB_1−287_, PknG_1−342_ or PknL_1−287_ and [γ-^33^P]-ATP and analysed by autoradiography. C. FtsZ phosphopeptides and phosphorylation sites identified by MALDI-TOF-MS and ESI-TOF-MS of tryptic digests of spot 3 in the 2D gel of the Δ*ppp* mutant and of tryptic digests after *in vitro* phosphorylation of FtsZ with PknA_1−287_ and PknL_1−287_. The FtsZ sequence coverage was between 35% and 45% and did not include all serine and threonine residues present in FtsZ. Unambiguously identified phosphorylation sites are shown in bold and underlined.

The abnormal cell morphology of the Δ*ppp* mutant indicated that one or several of the STPKs of *C. glutamicum* are involved in morphogenesis and cell division, as previously suggested for PknA and PknB (Fiuza *et al.*, 2008). Indeed, a 2D-PAGE comparison revealed that FtsZ, a key player in cell division (Kobayashi *et al.*, 1997; Honrubia *et al.*, 1998), is present in the wild type as one spot, whereas four spots differing in isoelectric point but not in molecular mass are detectable in the Δ*ppp* mutant (Fig. 8A). We therefore tested whether FtsZ can be phosphorylated by the kinase domains of PknA, PknB, PknG and PknL. As shown in Fig. 8B, FtsZ was phosphorylated by PknA_1−287_, PknB_1−287_ and PknL_1−287_ and thus identified as a novel substrate of these kinases. No phosphorylation of FtsZ was observed with the kinase domain of PknG.

In order to search for phosphorylation sites, tryptic digests of the FtsZ spots from 2D gels and FtsZ bands from 1D gels obtained after *in vitro* phosphorylation were analysed by MS. Three FtsZ phosphopeptides were detected after PknA_1−287_-dependent phosphorylation and four FtsZ phosphopeptides after PknL_1−287_-dependent phosphorylation by MALDI-TOF-MS and ESI-TOF-MS (Fig. 8C). The only phosphopeptide found in the analysed 2D gel spots was from spot 3 (Fig. 8A) and covered amino acid residues 381–395 (S_381_GGGLFT_387_T_388_GNDRDYR), which was also identified in the FtsZ protein phosphorylated by PknL_1−287_. Four phosphorylation sites could be identified by ESI-TOF-MS/MS, i.e. T108 in FtsZ phosphorylated by PknA and T63, S353 and T388 in FtsZ phosphorylated by PknL (Fig. 8C).

## Discussion

In our previous study we obtained evidence that the 2-oxoglutarate dehydrogenase inhibitor protein OdhI is phosphorylated *in vivo* not only by PknG, but also by one or several of the other three STPKs present in *C. glutamicum*, PknA, PknB and PknL. To determine which of these proteins can catalyse OdhI phosphorylation *in vivo*, the in-frame deletion mutants Δ*pknA*, Δ*pknB*, Δ*pknL*, Δ*pknAG*, Δ*pknAL*, Δ*pknBG*, Δ*pknBL*, Δ*pknLG*, Δ*pknALG* and Δ*pknBLG* were constructed. Together with the Δ*ppp* mutant, this set of mutant strains provides an excellent basis to analyse the *in vivo* function of the individual STPKs and of Ppp in *Corynebacterineae*. The fact that we obtained mutants lacking *pknA* or *pknB* either alone or together with *pknG* and/or *pknL* shows that none of these genes is essential in *C. glutamicum*. In a recent study, however, *pknA* and *pknB* were proposed to be essential in *C. glutamicum* (Fiuza *et al.*, 2008), similar to the situation reported for *M. tuberculosis* (Kang *et al.*, 2005; Fernandez *et al.*, 2006). Reasons for the differing results might be the use of different strains of *C. glutamicum* (ATCC 13032 and ATCC 13869) or the use of different methods for gene inactivation. In the case of strain ATCC 13869 it was tried to disrupt the genes by a single homologous recombination event leading to the genomic integration of a non-replicating plasmid containing internal fragments of either *pknA* or *pknB* (Fiuza *et al.*, 2008). The kanamycin promoter activity of this plasmid might inhibit expression of the upstream genes (*ftsI*, *ftsW*) and thereby cause the lethality of these constructs. We used a method in which the first homologous recombination event retains an intact copy of the gene to be disrupted and the second recombination event either restores the wild-type situation or generates a mutant in which the entire vector is removed and a few residual 5′- and 3′-codons of the deleted gene are fused by an introduced 21 bp DNA sequence. This in-frame deletion should have no effects on the expression of the up- and downstream genes.

The *in vivo* analysis of the OdhI phosphorylation status in the different STPK mutants by Western blot analysis and 2D gel electrophoresis indicated that besides PknG also PknA, PknB and PknL can contribute to OdhI phosphorylation, but the latter two only to a small extent. However, as the *in vivo* activity of the STPKs is presumably not constitutive, but controlled in response to yet unknown stimuli, the influence of these kinases on the OdhI phosphorylation status might vary under different experimental conditions. Regarding the *in vivo* phosphorylation sites of OdhI, we were able to confirm the existence of doubly phosphorylated OdhI and to show the simultaneous phosphorylation of T14 and T15. Moreover, hints for the phosphorylation of one or two additional residues of OdhI were obtained. The simultaneous phosphorylation of T14 and T15 and the occurrence of a triply phosphorylated OdhI may only be possible in the phosphatase-deficient Δ*ppp* mutant, but not in the wild type. In this case, the doubly phosphorylated OdhI detected in the wild type carries one phosphoryl group at T14 or T15 and the second one somewhere else in the C-terminal part of the protein. Studies are underway to confirm and identify the proposed additional phosphorylation site(s). The physiological function of a second phosphorylation of OdhI is unknown at present.

In a previous study it was reported that PknG activity is strictly dependent on its phosphorylation by PknA (Fiuza *et al.*, 2008). Several results presented here disagree with this statement. (i) The kinase domain of PknG (PknG_1−342_) isolated from *E. coli* showed autophosphorylation and OdhI transphosphorylation activity (Fig. 6B) and the same holds true for full-length PknG isolated from *E. coli* (data not shown). (ii) Full-length PknG_Strep_ isolated from the *C. glutamicum*Δ*pknA* mutant was capable of autophosphorylation and transphosphorylation of OdhI (Fig. S2). (iii) If PknG would require PknA to become active, the Δ*pknA* mutant should show the same growth defect on glutamine agar plates as the Δ*pknG* mutant, which is not the case (Fig. 2B). (iv) Different abundances of unphosphorylated and phosphorylated OdhI were observed in cell-free extracts of the Δ*pknA*, Δ*pknG* and Δ*pknAG* mutants by Western blot studies with OdhI antibodies and/or by 2D gel electrophoresis (Fig. 4). In the case of a PknA dependence of PknG, the abundances for the Δ*pknA* and the Δ*pknAG* mutant should be the same. In summary, our studies suggest that PknG is capable of autophosphorylation and OdhI phosphorylation independent of PknA. However, we do not exclude the possibility that *in vivo* transphosphorylation of PknG by PknA, PknB or PknL occurs and influences its activity.

In this work, the cell division protein FtsZ was identified as an *in vitro* substrate for PknA, PknB and PknL and as an *in vivo* substrate for Ppp in *C. glutamicum*. The presence of four FtsZ spots in the 2D gels of the Δ*ppp* mutant of which only the one with the highest pI value was detected in the wild type suggests that FtsZ can exist at least in mono-, di- and triphosphorylated forms *in vivo*. *In vitro* five different tryptic phosphopeptides were identified within FtsZ, covering residues 61–76, 92–119, 121–138, 325–368 and 381–395. The phosphopeptide 381–395 was also identified in one of the 2D gel spots, confirming that this peptide contains an *in vivo* phosphorylation site and T388 was shown to be phosphorylated by PknL *in vitro*. Furthermore, residues T63, T108 and S353 were identified as further phosphorylation sites. In a previous study it was reported that PknA of *M. tuberculosis* phosphorylates FtsZ *in vitro* and that the phosphorylated FtsZ shows a decreased GTPase activity resulting in a decreased polymerization activity (Thakur and Chakraborti, 2006). Phosphorylation of FtsZ from *C. glutamicum* might have a similar effect, which could be responsible for the abnormal morphology of the Δ*ppp* mutant, but the results of a protein sedimentation experiment similar to the one described by Thakur and Chakraborti (2006) were not consistent in our hands. Therefore, further studies are required to study the influence of phosphorylation on the properties and activities of FtsZ. It was shown previously that the reduced expression of *ftsZ* in *C. glutamicum* results in an abnormal cell morphology (Ramos *et al.*, 2005).

**Fig. 9:**
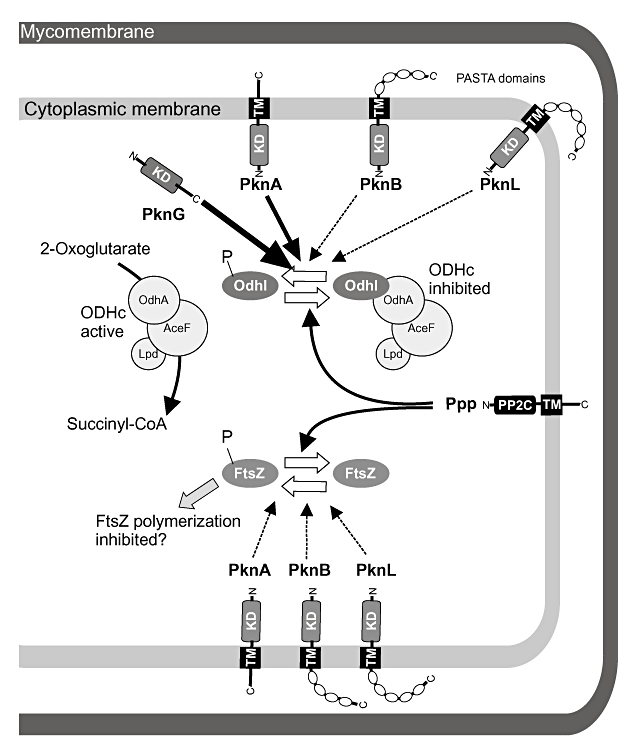
Model of STPK-dependent phosphorylation and Ppp-dependent dephosphorylation of OdhI and FtsZ in *C. glutamicum*.

The results presented here, which are summarized in Fig. 9, indicate that the STPKs of *C. glutamicum* have an overlapping substrate spectrum. In this way the activity of the target proteins OdhI and FtsZ can be controlled in response to the different, currently unknown stimuli of the STPKs. Whether this is a common situation will be explored in future studies aimed at the identification of further STPK substrates, their phosphorylation sites and the influence of phosphorylation on their activity.

## Experimental procedures

### Bacterial strains, media and culture conditions

The strains and plasmids used in this study are listed in Table S2. Strains used for cloning and overproduction of proteins were *E. coli* DH5α, *E. coli* BL21(DE3), *E. coli* BL21(DE3) containing pLysS (Studier and Moffatt, 1986), or *E. coli* BB1553 (Tomoyasu *et al.*, 2001). All *E. coli* strains were grown and maintained aerobically in Luria–Bertani (LB) medium at 37°C or 30°C (*E. coli* BB1553). For corynebacterial strain construction and maintenance, BHIS agar plates (brain–heart infusion agar from Difco Laboratories, Detroit, MI, USA with 0.5 M sorbitol) were used. *C. glutamicum* strains were cultivated aerobically in liquid brain–heart infusion medium (BHI) with 4% (w/v) glucose at 30°C. For growth experiments on agar plates with l-glutamine as sole carbon and nitrogen source, a modified CGXII medium (Keilhauer *et al.*, 1993) was used that lacked ammonium sulphate and urea and was supplemented with 1.5% agar, 30 mg l^−1^ 3,4-dihydroxybenzoic acid as iron chelator and 2% (w/v) l-glutamine. When required, media were supplemented with 100 μg ml^−1^ ampicillin, 25 μg ml^−1^ chloramphenicol, 50 μg ml^−1^ kanamycin (*E. coli*) or 25 μg ml^−1^ kanamycin (*C. glutamicum*).

### Standard recombinant DNA work

Oligonucleotides were obtained from Operon (Cologne, Germany) and are listed in Table S3. Enzymes used for DNA restriction, ligation or dephosphorylation were obtained from either Roche Diagnostics (Mannheim, Germany) or New England Biolabs (Frankfurt am Main, Germany). Routine methods like PCR, restriction or ligation were carried out according to standard protocols (Sambrook *et al.*, 2001). Chromosomal DNA from *C. glutamicum* was prepared as described (Eikmanns *et al.*, 1994). Plasmids from *E. coli* were isolated with the QIAprep Spin Miniprep Kit according to the manufacturer's instructions (Qiagen, Hilden, Germany). *E. coli* was transformed by the RbCl method (Hanahan, 1985) and *C. glutamicum* by electroporation (van der Rest *et al.*, 1999). DNA sequencing was performed with a Genetic Analyzer 3100-Avant (Applied Biosystems, Darmstadt, Germany). Sequencing reactions were carried out with the BigDye Terminator v3.1 Cycle Sequencing Kit (Applied Biosystems, Darmstadt, Germany).

### Construction of in-frame deletion mutants

In-frame deletion mutants of *C. glutamicum* lacking *pknA*, *pknB*, *pknG* and *pknL* were constructed via a two-step homologous recombination procedure as described (Niebisch and Bott, 2001). The up- and downstream regions (∼500 bp each) of *pknA* were amplified using *C. glutamicum* ATCC 13032 genomic DNA and the oligonucleotide pairs Δ*pknA*-1/Δ*pknA*-2 and Δ*pknA*-3/Δ*pknA*-4 respectively. The PCR products were subsequently fused by overlap extension PCR with the oligonucleotides Δ*pknA*-1/Δ*pknA*-4, resulting in PCR products of ∼1 kb. In the same way, the flanking regions of *pknB* and *pknL* were amplified and fused. The PCR fragments were digested with XmaI/SphI (*pknA*, *pknB*) or XbaI/SalI (*pknL*) and cloned into pK19*mobsacB* (Schäfer *et al.*, 1994), resulting in plasmids pK19*mobsacB*-Δ*pknA*, pK19*mobsacB*-Δ*pknB* and pK19*mobsacB*-Δ*pknL*. The PCR-derived fragments and the ligation sites were subjected to DNA sequence analysis and only plasmids without mutations were used further. Transfer of plasmids described above and pK19*mobsacB*-Δ*pknG* (Niebisch *et al.*, 2006) into *C. glutamicum* and screening for the first and second recombination event were performed as described (Niebisch and Bott, 2001). Kanamycin-sensitive and sucrose-resistant clones were tested by PCR analysis of chromosomal DNA with the oligonucleotides listed in Fig. S1 to distinguish between wild type and the desired mutants. In addition, the chromosomal deletions were confirmed in some cases by Southern blot analysis, which was performed as described (Niebisch and Bott, 2001). For this purpose, the chromosomal DNA was digested with BamHI and the respective digoxigenin-labelled overlap extension PCR product was used as probe (Fig. S1B). For construction of in-frame deletion mutants lacking two or three different STPK genes, the same procedure as described above was used and the deletions were verified by PCR (Fig. S1A).

### Overproduction and purification of the kinase domains of PknA, PknB, PknG, PknL and the phosphatase domain of Ppp

DNA fragments encoding the N-terminal regions including the kinase domains of PknA (residues 1–287), PknB (residues 1–287), PknG (residues 1–342), PknL (residues 1–287) and the N-terminal cytoplasmic region containing the phosphatase domain of Ppp (residues 1–309) were amplified by PCR using *C. glutamicum* ATCC 13032 genomic DNA as template and the oligonucleotide pairs *pknA*-1/2, *pknB*-1/2, *pknG*-1/2, *pknL*-1/2 and *ppp*-1/*ppp*-2 (Table S3). The resulting DNA fragments were digested with NdeI and XhoI and cloned into the expression vector pET16b cut with the same enzymes, leading to plasmids pET16b-*pknA*, pET16b-*pknB*, pET16b-*pknG*, pET16b-*pknL* and pET16b-*ppp*. The PCR-derived DNA portions and the ligation sites were subjected to DNA sequence analysis and only plasmids without mutations were used further. Plasmids pET16b-*pknB*, pET16b-*pknG*, pET16b-*pknL* and pET16b-*ppp* were transferred into *E. coli* BL21(DE3) and pET16b-*pknA* into *E. coli* BL21(DE3)/pLysS. The strains were grown at 37°C in 100 ml of LB medium containing 100 μg ml^−1^ ampicillin (and 25 μg ml^−1^ chloramphenicol in the case of the strain carrying pLysS) until they reached an OD_600_ of 0.5. Then overexpression of the target genes was induced by addition of 1 mM isopropyl β-d-thiogalactoside (IPTG) and the cultures were incubated for another 4 h at 30°C. Subsequently, the cells were harvested by centrifugation and stored at −20°C. For protein purification cells were thawed, washed once and re-suspended in 10 ml of TNI5 buffer (20 mM Tris/HCl pH 7.9, 500 mM NaCl and 5 mM imidazole). After addition of Complete EDTA-free protease inhibitor (Roche Diagnostics), the cell suspension was passed three times through a French pressure cell (SLM Aminco, Spectronic Instruments, Rochester, NY, USA) at 207 MPa. Intact cells and cell debris were removed by centrifugation (15 min, 5000 *g*, 4°C) and the cell-free extract was subjected to ultracentrifugation (1 h, 150 000 *g*, 4°C). The proteins PknA_1−287_, PknB_1−287_, PknG_1−342_, PknL_1−287_ and Ppp_1−309_ were present in the supernatant of the ultracentrifugation step and purified by Ni^2+^-chelate affinity chromatography using Ni-NTA superflow resin (Qiagen, Hilden, Germany). After washing the column with buffers TNI30, TNI50 and TNI100 (containing 30, 50 or 100 mM imidazole respectively), specifically bound proteins were eluted with TNI200 buffer (containing 200 mM imidazole). Protein-containing elution fractions were pooled and concentrated by ultrafiltration using Vivaspin columns with a molecular weight cut-off of 5000 (Sartorius Stedim Biotech, Aubagne Cedex, France). Subsequently, the elution buffer was exchanged against kinase buffer (25 mM Tris/HCl pH 7.5, 5 mM MgCl_2_, 2 mM MnCl_2_, 1 mM DTT) by gel filtration with PD10 columns (GE Healthcare) and the protein-containing fractions were again concentrated by ultrafiltration.

### Overproduction and purification of OdhI and FtsZ

OdhI containing a C-terminal *Strep*Tag-II was overproduced using the expression plasmid pAN3K-*odhI* described previously (Niebisch *et al.*, 2006) and either *E. coli* BL21(DE3) or *E. coli* BB1553 as host. Purification was achieved by affinity chromatography on *Strep*Tactin-Sepharose columns (IBA, Göttingen, Germany) as described before (Niebisch *et al.*, 2006). The *ftsZ* gene (encoding residues 1–442) was amplified by PCR from chromosomal DNA of *C. glutamicum* using the oligonucleotide pair *ftsZ*-1/2. The NdeI/XhoI-digested PCR product was cloned into the vector pET16b and checked by DNA sequence analysis. Plasmid pET16b-*ftsZ* was transferred into *E. coli* BL21(DE3)/pLysS and the strain was grown at 37°C in 500 ml of LB medium containing 50 μg ml^−1^ ampicillin and 25 μg ml^−1^ chloramphenicol until reaching an OD_600_ of 0.5. Then overexpression of FtsZ was induced by addition of 1 mM IPTG and the culture was incubated further for 3 h at 37°C. Subsequently the cells were harvested by centrifugation, washed and re-suspended in washing buffer (50 mM Tris/HCl pH 7.5, 100 mM NaCl, 5 mM MgCl_2_, 1 mM DTT, 10% glycerol) and after addition of Complete EDTA-free protease inhibitor (Roche Diagnostics), the cells were lysed by Fastprep (MP Biomedicals, Solon, OH, USA). Intact cells and cell debris were removed by centrifugation (30 min, 17 950 *g*, 4°C). FtsZ was present in the supernatant and purified by Ni^2+^-chelate affinity chromatography using Ni-NTA-Agarose (Qiagen, Hilden, Germany). Therefore, 0.5 ml of the Ni-NTA-Agarose was filled in 15 ml conical tubes (Falcon) and loaded with the cleared lysate. After incubation for 30 min at 4°C, samples were centrifuged for 4 min at 4°C at 805 *g*. The agarose was then washed first with washing buffer containing 15 mM imidazole and second with washing buffer containing 30 mM imidazole. After every step the agarose was centrifuged for 4 min at 4°C at 805 *g* and the supernatant was discarded. To eluate His-tagged FtsZ the imidazole concentration was increased to 500 mM imidazole. Protein-containing elution fractions were pooled and concentrated by ultrafiltration using Vivaspin columns (cut-off 5000 Da). Subsequently, the elution buffer was exchanged against kinase buffer by gel filtration as described above and protein-containing fractions were concentrated by ultrafiltration.

### Protein determination

Protein concentrations were determined with the bicinchoninic acid protein assay kit (Pierce Biotechnology, Rockford, IL, USA) or by measuring the absorbance at 280 nm with a NanoDrop 1000 Spectrophotometer (Thermo Fisher Scientific, Wilmington, DE, USA). The millimolar extinction coefficients used were 19.940 mM^−1^ cm^−1^ for PknA_1−287_, 10.555 mM^−1^ cm^−1^ for PknB_1−287_, 18.910 mM^−1^ cm^−1^ for PknG_1−342_, 10.430 mM^−1^ cm^−1^ for PknL_1−287_, 6085 mM^−1^ cm^−1^ for Ppp_1−309_, 6.990 mM^−1^ cm^−1^ for OdhI_Strep_ and 5.960 mM^−1^ cm^−1^ for FtsZ_His_.

### Analysis of the protein phosphorylation status by 2D gel electrophoresis and Western blot analysis

In order to analyse the phosphorylation status of OdhI and FtsZ *in vivo*, 10 ml of samples of cultures grown for 24 h in BHI medium containing 4% (w/v) glucose were centrifuged and the cells were washed and re-suspended in 1.0 ml of phosphate-buffered saline (PBS; 137 mM NaCl, 2.7 mM KCl, 4.3 mM Na_2_HPO_4_, 1.4 mM KH_2_PO_4_, pH 7.3) containing Complete EDTA-free protease inhibitor (Roche Diagnostics). After addition of 1 g of zirconia-silica beads (0.1 mm diameter; Roth, Karlsruhe, Germany) the cells were mechanically disrupted by 3 × 30 s bead beating. Intact cells and cell debris were removed by centrifugation at 5000 *g* for 15 min at 4°C and the cell-free extract was subjected to ultracentrifugation (1 h, 150 000 *g*, 4°C). Protein (300 μg) of the resulting supernatant was used for 2D gel electrophoresis as described previously (Schaffer *et al.*, 2001). Analysis of the OdhI phosphorylation status by Western blot analysis with OdhI antibodies was performed as described (Schultz *et al.*, 2007) using 20 μg protein of the ultracentrifugation supernatant for separation by SDS-PAGE. Relative quantification of the different phosphorylation states of proteins in 1D or 2D gels was performed by densitometry using a Fuji scanner and the software AIDA v2.41 (Fuji).

### Microscopic techniques

Cells for microscopy were grown in BHI medium with 4% (w/v) glucose to an OD_600_ of about 1.5. For phase-contrast microscopy, 1–3 μl of a culture sample was placed on a microscope slide that was coated with a thin 1.5% agarose layer and covered by a coverslip. Images were taken on a Zeiss AxioImager M1 that was equipped with a Zeiss AxioCam HR3 camera. An EC Plan-Neofluar 100-magnification, 1.3-numeric-aperture oil immersion Ph3 objective was used. Digital images were acquired and analysed with AxioVision 4.6 software (Zeiss, Göttingen, Germany).

### *In vitro* kinase and phosphatase assays

For *in vitro* phosphorylation, 20 μl of reaction mixtures containing 2–5 μg of PknA_1−287_, PknB_1−287_, PknG_1−342_ or PknL_1−287_ and 2 mM non-radioactive ATP in kinase buffer (see above) were incubated for 1 h at 37°C. For testing the phosphorylation of OdhI and FtsZ, the mixtures contained 2 μg of OdhI or 2 μg of FtsZ for subsequent analysis by SDS-PAGE or 0.05 μg of OdhI protein for Western blot analysis. *In vitro* phosphorylation with [γ-^33^P]-ATP (1 μCi) was carried out for 30 min at 37°C. The reaction was stopped by addition of SDS sample buffer and the mixture was heated at 98°C for 10 min before SDS-PAGE and autoradiography.

For *in vitro* dephosphorylation, a 120 μl reaction mastermix containing 18 μg of purified phosphatase Ppp_1−309_ and 12 μg of OdhI_Strep_ purified from strain *C. glutamicum*Δ*ppp*/pJC1-*odhI* was incubated in kinase buffer (see above). After 1, 5, 10, 15, 30 and 120 min 20 μl aliquots were taken and immediately mixed with SDS sample buffer. Subsequently, the samples were heated for 10 min at 98°C and analysed by SDS-PAGE.

### MS analysis

Identification of proteins from Coomassie-stained 1D or 2D SDS-polyacrylamide gels and search for phosphopeptides were performed by peptide mass fingerprinting of tryptic digests as described by Schaffer *et al.* (2001), except that peptides were extracted by addition of 0.2% (v/v) trifluoroacetic acid in 30% (v/v) acetonitrile instead of 0.1% (v/v) trifluoroacetic acid in 30% (v/v) acetonitrile. MALDI-TOF-MS was performed with an Ultraflex III TOF/TOF mass spectrometer (Bruker Daltonics, Bremen, Germany). The MASCOT software (Perkins *et al.*, 1999) was used to compare the peptide mass patterns obtained with those of all proteins from the theoretical *C. glutamicum* proteome. The molecular weight search (MOWSE) scoring scheme (Pappin *et al.*, 1993) with a cut-off value of 50 was used for unequivocal identification of proteins. The FtsZ phosphorylation sites were identified by ESI-MS/MS analysis. These experiments were performed with an ESI-QqTOF hybrid mass spectrometer (QSTAR XL, Applied Biosystems) equipped with a nanoflow electrospray source. Sequence analysis and peptide assignment were accomplished using the GPMAW software (http://www.welcome.to/gpmaw).
