# Supplementary material for: Genetic and biochemical analysis of the serine/threonine protein kinases PknA, PknB, PknG and PknL of Corynebacterium glutamicum: evidence for non-essentiality and for phosphorylation of OdhI and FtsZ by multiple kinases
Source: Mol Microbiol. 2009 Oct 13;74(3):724–41. doi: 10.1111/j.1365-2958.2009.06897.x (PMC2784874; doi:10.1111/j.1365-2958.2009.06897.x)
Supplement: Supplementary file 1 [file mmi0074-0724-SD1.pdf]

**Table S1.** *In vivo* OdhI phosphorylation state as determined by Western blot analysis and 2D gel analysis of cell-free extracts of the indicated *C. glutamicum* strains. The strains were cultivated in triplicate for 24 h in BHI medium with 4% (w/v) glucose and 20 µg cell extract protein was used for Western blot analysis with OdhI antibodies or 300 µg for 2D gel analysis. The percentage (mean values with standard deviation) of unphosphorylated OdhI and phosphorylated OdhI was calculated by densitometric analysis.

| Strain | Western blot analysis | | 2D gel analysis | | | |
|---|---|---|---|---|---|---|
| | unphosphor. OdhI | phosphoryl. OdhI | unphosphor. OdhI | phosphoryl. OdhI | monophosph. OdhI | diphosphor. OdhI |
| Wild type | 66 ±5 | 34 ±5 | 49 ±7 | 51 ±7 | 49 ±5 | 3 ±3 |
| Δ*ppp* | 3 ±4 | 97 ±4 | 4 ±1 | 96 ±1 | 50 ±13 | 46 ±13 |
| Δ*pknG* | 83 ±11 | 17 ±11 | 66 ±13 | 34 ±13 | 30 ±12 | 4 ±2 |
| Δ*pknA* | 77 ±9 | 23 ±9 | n. d. | n. d. | n. d. | n. d. |
| Δ*pknB* | 64 ±6 | 36 ±6 | n. d. | n. d. | n. d. | n. d. |
| Δ*pknL* | 61 ±5 | 39 ±5 | n. d. | n. d. | n. d. | n. d. |
| Δ*pknAG* | 92 ±7 | 8 ±7 | 76 ±10 | 24 ±10 | 16 ±6 | 7 ±4 |
| Δ*pknBG* | 87 ±10 | 13 ±10 | 72 ±11 | 28 ±11 | 26 ±11 | 2 ±1 |
| Δ*pknLG* | 87 ±12 | 13 ±12 | 66 ±11 | 34 ±11 | 29 ±8 | 5 ±3 |
| Δ*pknAL* | 70 ±6 | 30 ±6 | n. d. | n. d. | n. d. | n. d. |
| Δ*pknBL* | 63 ±7 | 37 ±7 | n. d. | n. d. | n. d. | n. d. |
| Δ*pknAGL* | 91 ±6 | 9 ±6 | 87 ±5 | 13 ±5 | 9 ±7 | 4 ±3 |
| Δ*pknBGL* | 95 ±3 | 5 ±3 | 80 ±8 | 20 ±8 | 16 ±6 | 4 ±2 |

**Table S2**. Bacterial strains and plasmids used in this study.

| Strains | Relevant characteristics | Source or reference |
|---|---|---|
| *E. coli* strains | | |
| DH5α | *supE44* Δ*lacU169* (Φ80*lacZ*ΔM15) *hsdR17 recA1 endA1 gyrA96 thi-1 relA1* | Invitrogen |
| BL21 (DE3) | F–*ompT* $hsdS_B$($r_B^-m_B^-$) *gal dcm* (DE3) | (Studier and Moffatt, 1986) |
| BL21 (DE3) pLysS | F–*ompT* $hsdS_B$($r_B^-m_B^-$) *gal dcm* (DE3); contains plasmid pLysS (Cam$^{R.}$) | (Studier and Moffatt, 1986) |
| BB1553 | MC4100 Δ*dnaK::cat sidB1* | (Tomoyasu *et al.*, 2001) |
| *C. glutamicum* strains | | |
| ATCC13032 | Biotin-auxotrophic wild type strain | (Kinoshita *et al.*, 1957) |
| Δ*ppp* | Wild type derivative with in-frame deletion of *ppp* (cg0062) | (Schultz *et al.*, 2007) |
| Δ*pknA* | Wild type derivative with in-frame deletion of *pknA* (cg0059) | This work |
| Δ*pknB* | Wild type derivative with in-frame deletion of *pknB* (cg0057) | This work |
| Δ*pknG* | Wild type derivative with in-frame deletion of *pknG* (cg3046) | (Niebisch *et al.*, 2006) |
| Δ*pknL* | Wild type derivative with in-frame deletion of *pknL* (cg2388) | This work |
| Δ*pknAL* | Δ*pknA* derivative with additional in-frame deletion of *pknL* | This work |
| Δ*pknBL* | Δ*pknB* derivative with additional in-frame deletion of *pknL* | This work |
| Δ*pknAG* | Δ*pknA* derivative with additional in-frame deletion of *pknG* | This work |
| Δ*pknBG* | Δ*pknB* derivative with additional in-frame deletion of *pknG* | This work |
| Δ*pknLG* | Δ*pknL* derivative with additional in-frame deletion of *pknG* | This work |
| Δ*pknALG* | Δ*pknAL* derivative with additional in-frame deletion of *pknG* | This work |

| | | |
|---|---|---|
| Δ*pknBLG* | Δ*pknBL* derivative with additional in-frame deletion of *pknG* | This work |
| Plasmids | | |
| pAN3K-*odhI* | $Kan^R$; derivative of the *E. coli* expression vector pASK-IBA3C (IBA, Göttingen, Germany) for anhydrotetracycline-inducible production of C-terminally *Strep*-tagged proteins and allowing replication and expression in *C. glutamicum*; contains the *C. glutamicum* wild type *odhI* gene (*cg1630*) | (Niebisch *et al.*, 2006) |
| pJC1-*odhI* | $Kan^R$; pJC1 derivative containing the *C. glutamicum* wild-type *odhI* gene with its native promoter and a *Strep*Tag-II coding sequence before the *odhI* stop codon | (Niebisch *et al.*, 2006) |
| pET16b | $Amp^R$; vector for overexpression of genes in *E. coli*, adding an N-terminal decahistidine tag to the synthesized protein (pBR322 $oriV_{E.c.}$, $P_{T7}$, *lacI*) | Novagen |
| pET16b-*pknA* | pET16b derivative for overproduction of $PknA_{1-287}$ | This work |
| pET16b-*pknB* | pET16b derivative for overproduction of $PknB_{1-287}$ | This work |
| pET16b-*pknG* | pET16b derivative for overproduction of $PknG_{1-342}$ | This work |
| pET16b-*pknL* | pET16b derivative for overproduction of $PknL_{1-287}$ | This work |
| pET16b-*ftsZ* | pET16b derivative for overproduction of $FtsZ_{1-442}$ (cg2366) | This work |
| pET16b-*ppp* | pET16b derivative for overproduction of $Ppp_{1-309}$ (cg0062) | This work |
| pK19*mobsacB* | $Kan^R$; *E. coli* vector for generating *C. glutamicum* deletion mutants | (Schäfer *et al.*, 1994) |
| pK19*mobsacB*-Δ*pknA* | pK19*mobsacB* derivative containing an overlap extension PCR product obtained with oligonucleotides Δ*pknA*-1-4 that covers the flanking regions of the *pknA* gene | This work |
| pK19*mobsacB*-Δ*pknB* | pK19*mobsacB* derivative containing an overlap extension PCR product obtained with oligonucleotides Δ*pknB*-1-4 that covers the flanking regions of the *pknB* gene | This work |

| | | |
|---|---|---|
| pK19*mobsacB*-Δ*pknG* | pK19*mobsacB* derivative containing an overlap exension PCR product that covers the flanking regions of the *pknG* gene | (Niebisch *et al.*, 2006) |
| pK19*mobsacB*-Δ*pknL* | pK19*mobsacB* derivative containing an overlap extension PCR product obtained with oligonucleotides Δ*pknL*-1-4 that covers the flanking regions of the *pknL* gene | This work |
| pEKEx2-*pknG* | Kan$^{R}$; *C. glutamicum* expression vector for IPTG-inducible gene expression; contains the *C. glutamicum pknG* gene with the native ribosome-binding site and a *Strep*Tag-II coding sequence before the *pknG* stop codon | (Niebisch *et al.*, 2006) |

**Table S3.** Oligonucleotides used in this work. Restriction sites are underlined and complementary sequences of oligonucleotide pairs used for overlap extension PCR are shown in italic.

| Name | Sequence (5´→ 3´) | Restriction enzyme |
|---|---|---|
| Δ*pknA*-1 | ATATCCCGGGCTACACCGGAATTCAGATCG | XmaI |
| Δ*pknA*-2 | *CCCATCCACTAAACTTAAACA*AGCCCGCTTGCGCTT CATTGC | |
| Δ*pknA*-3 | *TGTTTAAGTTTAGTGGATGGG*GACGCCGTAAATGG CCTATTGG | |
| Δ*pknA*-4 | TGAGCATGCGGAGGTTTGAGTCATGGC | SphI |
| Δ*pknB*-1 | TGAGCATGCTGTGCGCCTTGGCAAGCG | SphI |
| Δ*pknB*-2 | *CCCATCCACTAAACTTAAACA*GCGATCAGCGATCA CGAAGGTC | |
| Δ*pknB*-3 | *TGTTTAAGTTTAGTGGATGGG*CGACTCTTCGAATT CGATCTCGC | |
| Δ*pknB*-4 | ATATCCCGGGCGCTGTGCTGCGGCAGTCAC | XmaI |
| Δ*pknL*-1 | GACTCTAGAAAGCGCTTAGAACGCCATTG | XbaI |
| Δ*pknL*-2 | *CCCATCCACTAAACTTAAACA*GTCACCGACCTTCA AGTTTGCC | |
| Δ*pknL*-3 | *TGTTTAAGTTTAGTGGATGGG*GAAGTCTCGGTAG GGGGAGAAG | |
| Δ*pknL*-4 | TGAGTCGACGCCTGAATGGCGCGTGCTG | SalI |
| Δ*pknA*-for | GGAAGCCTCAGAGCGCAATAC | |
| Δ*pknA*-rev2 | CGACCACACCGAGAGAATAA | |
| Δ*pknB*-for | AACTGCCAAACGAGAGTGCC | |
| Δ*pknB*-rev2 | ACATCGTCGAAGAAGCCAGC | |
| Δ*pknG*-for | GCTGCGCGGTTTTGAAGTGG | |
| Δ*pknG*-rev | GATCGATCCAGAGCGTAACGC | |
| Δ*pknG*-rev2 | GAAGTTGTAGGCCTTCACGA | |
| Δ*pknL*-for | CATCGTTACGGTGATCATCG | |
| Δ*pknL*-rev | GGATGCTAATGGATGGTCGG | |
| Δ*pknL*-rev2 | GTGAGCAGCTCAAAGAGCAC | |
| *pknA*-1 | ATGCCATATGAGTCAAGAAGACATCAC | NdeI |
| *pknA*-2 | ATGCCTCGAGCGGGCGCTTGCCAAGGCGCA | XhoI |
| *pknB*-1 | ATGCCATATGGTGACCTTCGTGATCGCTGA | NdeI |
| *pknB*-2 | ATGCCTCGAGTGCGGCATGGGAGACTGCAT | XhoI |
| *pknG*-1 | ATGCCATATGAAGGATAATGAAGATTT | NdeI |
| *pknG*-2 | ATGCCTCGAGTACTTCGGGTGCTTGGAATC | XhoI |
| *pknL*-1 | ATGCCATATGGCAAACTTGAAGGTCGG | NdeI |
| *pknL*-2 | ATGCCTCGAGAACCGGCACAGGGACCCGGA | XhoI |
| *ftsZ*-1 | CAGCATATGACCTCACCGAACAACTAC | NdeI |
| *ftsZ*-2 | CAGCTCGAGTTACTGGAGGAAGCTGGG | XhoI |
| *ppp-1* | ATGCCATATGTTGACACTTAAATATGC | NdeI |
| *ppp-2* | ATGCCTCGAGTTTGCTGGAACTTTTCTCTG | XhoI |

A

| Lane | Gene analysed | Primer combination | Expected fragment size: Wild type | Mutant |
|---|---|---|---|---|
| A1 | *pknA* | ΔpknA-for + ΔpknA-rev2 | 881 bp | no product |
| B1 | *pknB* | ΔpknB-for + ΔpknB-rev2 | 1260 bp | no product |
| G1 | *pknG* | ΔpknG-for + ΔpknG-rev2 | 1400 bp | no product |
| L1 | *pknL* | ΔpknL-for + ΔpknL-rev2 | 1320 bp | no product |
| A2 | *pknA* | ΔpknA-for + ΔpknA-4 | 2161 bp | 1029 bp |
| B2 | *pknB* | ΔpknB-for + ΔpknB-1 | 3006 bp | 1027 bp |
| G2 | *pknG* | ΔpknG-for + ΔpknG-rev | 3764 bp | 1364 bp |
| L2 | *pknL* | ΔpknL-for + ΔpknL-rev | 3640 bp | 1502 bp |

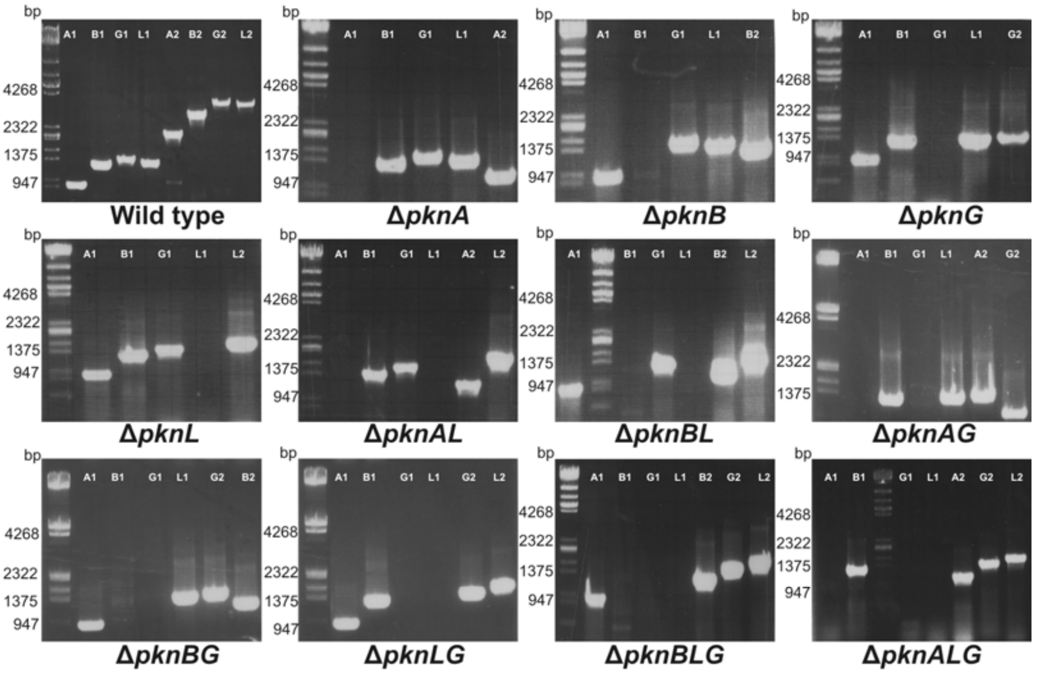


B

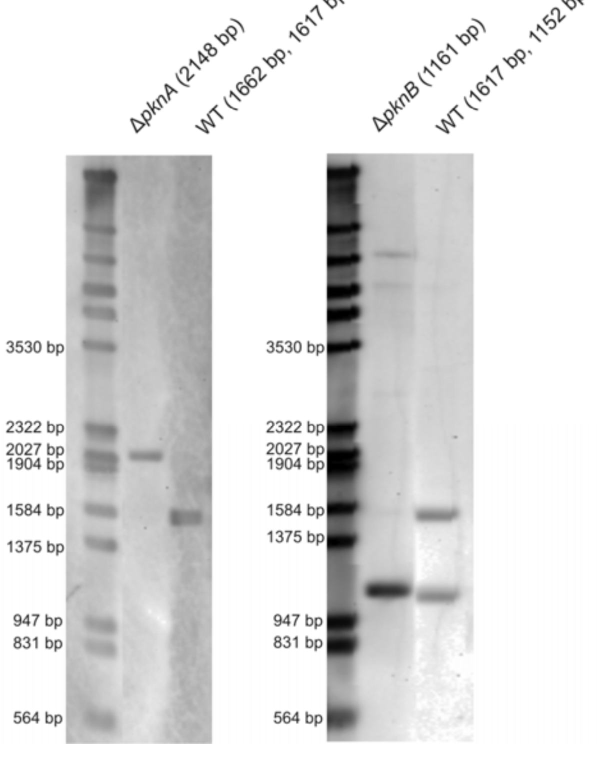


17

**Fig. S1.** Verification of the deletions of *pknA*, *pknB*, *pknL*, and *pknG* in the different single, double and triple mutants of *C. glutamicum* used in this study. (A) PCR analysis using chromosomal DNA of the indicated strains and the oligonucleotide pairs shown. The sizes of the expected PCR products are indicated. Each deletion was analysed with two primer pairs, one that results in a PCR product and another one that gives no PCR product in case of a successful deletion. (B) Southern blot analysis used to confirm the deletion of *pknA* and *pknB*. Chromosomal DNA of the wild type and mutant strains Δ*pknA* and Δ*pknB* was digested with BamHI and probed with the 1-kb DIG-labelled overlap extension PCR products used for construction of pK19*mobsacB*-Δ*pknA* and pK19*mobsacB*-Δ*pknB*. In the case of *pknA*, hybridizing fragments of 1617 bp and 1662 bp were expected for the wild type and only one 2148 bp fragment for the Δ*pknA* mutant. In the case of *pknB*, two fragments (1152 bp, 1617 bp) were expected for the wild type and one fragment of 1161 bp for the Δ*pknB* mutant.

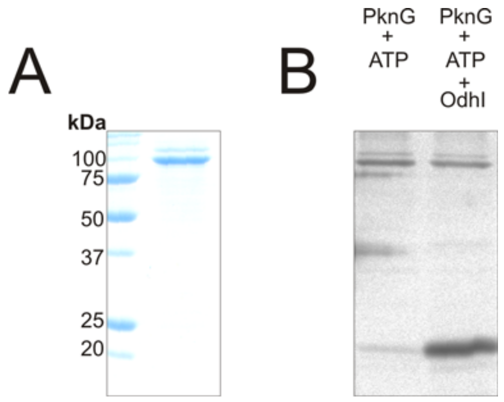


**Fig. S2.** PknG autophosphorylation and transphosphorylation of OdhI.

A. Coomassie-stained SDS-polyacrylamide gel showing molecular mass standards (lane 1) and $PknG_{Strep}$ purified by *Strep*Tactin affinity chromatography from cells of *C. glutamicum* Δ*pknA* containing plasmid pEKEx2-*pknG* (lane 2).

B. Autoradiogram of samples containing purified $PknG_{Strep}$ (1.5 µg) and 1 µCi [$\gamma$–$^{33}$P]-ATP (lane 1) or $PknG_{Strep}$ (1.5 µg), 1 µCi [$\gamma$-$^{33}$P]-ATP and 2 µg $OdhI_{Strep}$ (lane 2). Before SDS-PAGE, the samples were incubated for 30 min at 37°C.
